# Gene Therapy for Cardiovascular Disease: Basic Research and Clinical Prospects

**DOI:** 10.3389/fcvm.2021.760140

**Published:** 2021-11-05

**Authors:** Genmao Cao, Xuezhen Xuan, Ruijing Zhang, Jie Hu, Honglin Dong

**Affiliations:** ^1^Department of Vascular Surgery, The Second Hospital of Shanxi Medical University, Taiyuan, China; ^2^Department of Nephrology, The Second Hospital of Shanxi Medical University, Taiyuan, China

**Keywords:** gene therapy, cardiovascular disease, ethical concerns, delivering system, gene-editing technology

## Abstract

In recent years, the vital role of genetic factors in human diseases have been widely recognized by scholars with the deepening of life science research, accompanied by the rapid development of gene-editing technology. In early years, scientists used homologous recombination technology to establish gene knock-out and gene knock-in animal models, and then appeared the second-generation gene-editing technology zinc-finger nucleases (ZFNs) and transcription activator–like effector nucleases (TALENs) that relied on nucleic acid binding proteins and endonucleases and the third-generation gene-editing technology that functioned through protein–nucleic acids complexes—CRISPR/Cas9 system. This holds another promise for refractory diseases and genetic diseases. Cardiovascular disease (CVD) has always been the focus of clinical and basic research because of its high incidence and high disability rate, which seriously affects the long-term survival and quality of life of patients. Because some inherited cardiovascular diseases do not respond well to drug and surgical treatment, researchers are trying to use rapidly developing genetic techniques to develop initial attempts. However, significant obstacles to clinical application of gene therapy still exists, such as insufficient understanding of the nature of cardiovascular disease, limitations of genetic technology, or ethical concerns. This review mainly introduces the types and mechanisms of gene-editing techniques, ethical concerns of gene therapy, the application of gene therapy in atherosclerosis and inheritable cardiovascular diseases, in-stent restenosis, and delivering systems.

## Introduction

Genome editing technologies are continually emerging and evolving in recent years, leading to fundamental upgrades of the biomedical research model. In the early years, scientists used homologous recombination technology to establish animal models of gene knock-out and gene knock-in mutations. With the advent of the second generation of gene-editing technology ZFNs and TALENs which relied on nucleic acid-binding proteins and endonuclease and the third generation of gene-editing technology clustered regularly interspaced short palindromic repeats (CRISPR)/CRISPR-associated protein 9 (Cas9) system which functions through protein nucleic acid complex, researchers could achieve falling off-target incidence, improving editing efficiency, and expanding application scope.

Cardiovascular diseases refer to a class of heart or artery-related disease of the host, such as coronary artery disease, stroke, peripheral arterial disease, cardiomyopathy, aortic aneurysm, hypertensive heart disease, rheumatic heart disease, etc. (1). Non-traumatic arterial disease is usually characterized by inflammation, stenosis, and occlusion of the arteries, followed by insufficient blood supply and loss of function of the target organs. Artery aneurysms are anomalous dilated arteries that are prone to rupture spontaneously.

At present, nearly 10,000 diseases have been found to be hereditary, among which more than 100 monogenic inherited cardiovascular diseases are accounted for (2). Monogenic inherited cardiovascular diseases are cardiovascular diseases caused by single gene mutation and conformed to the Mendelian genetic law, such as Marfan syndrome, familial pulmonary hypertension, etc. The clinical manifestations of these diseases are usually catastrophic and tend to be familial clustering (3). Emerging clinical trials and animal experiments have confirmed the possibility of gene-editing technology in treating single-gene diseases. Applying gene-editing technology to prevent and treat cardiovascular diseases, especially congenital artery diseases, has become the focus of current cardiovascular research and guide the future direction of therapeutic approaches.

## Ethical Concerns

This review discussed technical possibilities of gene therapy for severe cardiovascular disease. Early gene therapy involves transferring gene-packaged vectors to supplement a missing function or protein. The recent emergence of CRISPR technology and base editor (BE) achieved higher feasibility for making gene-editing efficient and convenient. However, as an important research tool, gene-editing has never been distanced from ethical debate since the concept emerged.

The ethical problems are shared in different aspects in terms of clinical therapy on human and experimental research in animals. It is generally considered that ethical limits on gene therapy should adjust with the scope of gene modification in human. Based on the levels of alternation in genes, herein presented three categories of gene therapy: (1) Somatic non-integrated gene-replacement therapy; (2) somatic gene-editing therapy; (3) Germline gene-editing therapy.

### Ethical Concerns of Gene Therapy for Human

#### Somatic Non-integrated Gene-Replacement Therapy

Somatic non-integration gene therapy can transfer copies of normally functioning genes (or the coding sequence, cDNA) into dysfunctional cells to compensate, hence being a treatment for monogenic diseases (4). After being incorporated into somatic cells, foreign genes express independently from somatic genome. Thus, inadvertent genetic manipulation in genome, especially in germline cell, should be unconditionally prevented (5). Even though somatic gene replacement can increase the long-term survival and fertility of the victim, but such genetic disorders will be inherited by next generation through reproduction, therefore the proportion of gene defects in total population increased (4).

#### Somatic Gene-Editing Therapy

Somatic gene-editing therapy can permanently change target gene with endonuclease through gene disruption, gene deletion, gene insertion, gene replacement, and nucleotides substitution (6). It can be viewed as an advanced version of non-integrated gene-replacement therapy, as it avoids the DNA insertion and eliminates the generation of erroneous products by inducing gene mutation *in situ* (7).

In 2017, Committee on Human Gene Editing: Scientific, Medical, and Ethical Considerations (established by National Academy of Sciences and National Academy of Medicine) published a report entitled “*Human Genome Editing: Science, Ethics, and Governance*” (8). The report recommended somatic gene-editing therapy authorized as treatment or prevention only for disease or disability. In practice, platform technology, cell type, target genomic location, and other factors should be comprehensively taken into consideration to weigh the risk and benefit. Besides, disclosed and inclusive public debates should be conducted before approval for clinical trials.

#### Germline Gene-Editing Therapy

Germline gene-editing therapy can correct pathogenic mutations in gametes or embryos and further cutting off the inheritance of severe genetic diseases (9). The risks of somatic gene-editing are highly outweighed its benefit in treatment and long-term efficacy. A few risks needs taking into consideration in germline gene-editing: (1) any insertion/indels mutation in germline cells can cause unpredictable changes in next generation; (2) the informed consent from the next generation is impossible to be obtained (10). In fact, for most monogenic diseases, assisted reproductive technology (ART) utilizing *in vitro* fertilization (IVF) and pre-implantation genetic diagnosis (PGD) is capable enough to avoid the bequeathal of disease (11). However, when both parents are homozygous for recessive monogenic disorder, or one parent are homozygous for dominant monogenic disorder, the heritability will be 100% and germline gene-editing might be the only solution under the circumstances.

In 2020, International Commission on the Clinical Use of Human Germline Genome Editing (established by National Academy of Sciences and National Academy of Medicine) published a consensus study report entitled “*Heritable Human Genome Editing,”* which defined a practical and comprehensive guideline for clinical application of Heritable Human Genome Editing (HHGE) (12). The report classified clinical application areas of HHGE into six categories (see Table 1). According to the report, HHGE is suitable for all severe monogenic diseases with 100% heritability and a subset of severe monogenic diseases with 25–50% heritability. The report also recommended that any HHGE in clinical practice should require a detailed protocol, informed consent, and long-term monitoring on efficacy.

**Table 1:** Category of applications of HHGE and the recommendation from International Commission on the Clinical Use of Human Germline Genome Editing.

**Category**	**Definition**	**Characteristics**	**Recommendation**
A	Serious monogenic Diseases Heritability: 100%	◆ Autosomal dominant disease (one parent carries affected homozygote) ◆ Autosomal recessive disease (both parents carry affected homozygotes) ◆ X-linked recessive diseases (female parent carries affected homozygote, male parent carries affected hemizygote)	All is suitable for HHGE
B	Serious monogenic Diseases Heritability: 25–50%	◆ Autosomal dominant disease (one parent carries affected heterozygote) ◆ Autosomal recessive disease (both parents carry affected heterozygotes) ◆ X-linked dominant disease (female parent carries affected heterozygote) ◆ X-linked recessive diseases (female parent carries affected heterozygote, male parent carries affected hemizygote)	A small subset is suitable for HHGE
C	Mild monogenic diseases	Mainly affecting quality of life; Could be mitigated by medical or lifestyle interventions e.g., familial hypercholesterolemia	Unsuitable for HHGE, because the balance of risks and benefits is unknown
D	Polygenic diseases	Disease were caused by a large number of genetic variants e.g., T2DM	
E	Other applications	Enhancing human ability, or obtaining new function	
F	Monogenic conditions that cause infertility	Treating e infertility caused by monogenic variant in germline cell	

*HHGE, Heritable Human Genome Editing*.

HHGE can effectively prevent the inheritance of monogenic disease due to the accurate and efficient genome editing. However, evidences of HHGE in complex polygenic diseases are far insufficient. Currently, no technique can completely control DNA repair after double strand break (DSB), and no analytical method could comprehensively evaluate the efficiency and off-target effects of human gene-editing.

In terms of economic benefits, the huge cost of gene therapy in production, transportation, storage, clinical implementation, and post-treatment monitoring makes it an uncoverable burden for health insurance almost in every country. Another restriction is the unclear pathogenic genes and mechanisms involved in phenotypes. For instance, the onset of atherosclerosis involves multiple pathways and genes. Current animal experiments have tested modifications on several targets, but the ideal target gene has not been determined yet. In conclusion, the ethical restriction of gene therapy should factor in the severity of disease, the benefits for patients, and all potential risks.

### Ethical Concerns of Gene Therapy Research on Animals

Some scholars claim the unnecessarity of validating CRISPR technique in animals such as mice and primates due to differences in gene expression between humans and animals (13). For HHGE, direct experiments on human embryos seem necessary, considering the discrepancies in cellular DNA repair mechanisms as well as in early embryonic development among species. As the most popular gene-editing technique, although CRISPR/Cas9 has improved comparing to the former generations, the defects of low efficiency and low specificity still concerns (7). Undesired off-target effects may cause unknown phenotypic changes (14). And persistent off-target effects could trigger pathogenic editing, toxic substances, or cell cancerization, which frequently occurred in early experiments on animals.

## Gene-Editing Technology

### Zinc-Finger Nucleases (ZFNs)

ZFNs are a kind of artificially synthesized restriction endonucleases. The zinc finger DNA-binding domain was fused with the DNA cleavage domain of restriction enzymes (15). The researchers engineered ZFNs' DNA binding domain to target different DNA sequences, allowing ZFNs to bind to target sequences in complex genomes and perform specific cleavage from the DNA cleavage domain (16).

**Figure 1:**
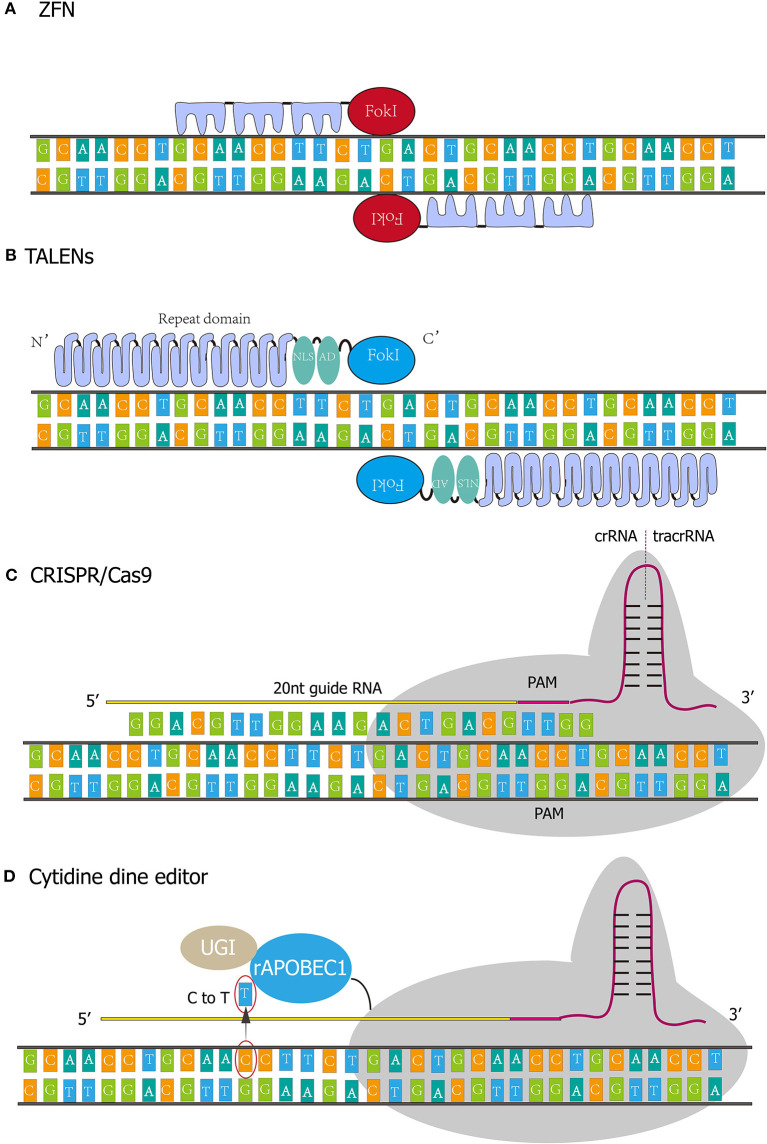
Four kinds of gene-editing technologies. **(A)** The DNA binding domain of the Zinc finger generally contains three independent Zinc finger (ZF) repeats, and each ZF repeat recognizes three consecutive bases. **(B)** TALENs comprise translocation activation domain (AD), DNA binding domain, nuclear localization signals (NLS), and nuclease domain. Every repeat in DNA binding domain corresponds to nucleotides in a one-to-one relationship. **(C)** CRISPR/Cas9 system functions through protein nucleic acid complex. The whole system incorporates crRNA, tracrRNA, and Cas9 protein. crRNA contains single guide RNA (20 nt) and PAM sequence (NGG). **(D)** rAPOBEC1 is a cytidine deaminase that deaminates cytosine to uracil and then the uracil will be replaced by thymine. Uracil DNA glycosylase inhibitor (UGI) could elevate editing efficiency by inhibiting reversing the U-G pair to the original C-G pair.

ZFNs were initially found in the observation on FokI by Chandrasegaran (17). This is a natural IIS type restriction enzyme with a recognition region and cleavage structure. No specificity of the cleavage region was observed, and the cleavage site can be redirected by replacing the original recognition region (18, 19). Taking Cys2His2 Zinc finger as an example, its structure consists of a Zinc atom wrapped in ~30 amino acids. The DNA binding domain of the Zinc finger generally contains three independent Zinc finger (ZF) repeats, and each ZF repeat recognizes three consecutive bases (Figure 1A) (20). In 1998, Bitinaite et al. found that DNA cleavage domains had to function in the form of dimer due to the weak binding ability of cleavage domains to DNA chains (21). Subsequent studies showed that when constructing ZF nuclease, two ZFNs should be designed for the adjacent regions of each DNA chain. The DNA cleavage domain can be located at the exact position of the double-strand, to achieve the best cleavage effect. There is a spacer structure called “spacer zone” between two ZFNs. The length of this structure is 5~6 bp, and even 7 bp can work typically. Only a reasonable “spacer zone” design can ensure that the ZFNs dimer has the best workspace (22).

ZFNs is reputed to be the earliest artificial genome editing technology. After ZFNs is designed and synthesized according to the target gene sequence, DNA could be precisely cut to form DSB. Then target gene was deactivated by breaking non-homologous end joining (NHEJ) or was entirely repaired by homologous recombination (HR) (23).

The primary defect of ZFNs-mediated gene-editing is that DNA cutting by ZFNs requires the dimerization of two FokI cutting regions and requires at least one recognition unit to bind DNA (24). Although the DNA recognition domain has a solid specific recognition ability, the cutting process of ZFNs is not entirely dependent on the formation of the heterodimer. Therefore, the formation of the homodimer is likely to cause off-target effect and eventually may lead to DNA mismatch and sequence change, resulting in serve cytotoxicity (25). When off-target effects accumulate to a certain level and beyond the cell's self-repair ability threshold, cell apoptosis will occur. On the other hand, this technique is still limited by existing biological research methods, so it is difficult to predict the precision and consequences of the intracellular operation. If ZFNs causes mutations in non-target genes, it may lead to a series of catastrophic consequences, especially in human application. In addition, among several gene-editing techniques, ZFNs is more likely to trigger immune response *in vivo*. Given existing technical merit, it is impossible to predict whether the introduced ZFNs protein will induce the immune system's attack. Currently, ZFNs technology can only be applied to *in vitro* operations. Extracted cells were edited *in vitro* and then infused back into the patient, while the direct introduction of related ZFNs elements into the patient's body for *in vivo* gene-editing has more significant potential risks and lower efficiency (26).

### Transcription Activator–Like Effector Nucleases (TALENs)

TALENs were assembled by transcription activator-like effectors (TALEs) and nucleases. TALEs were first discovered in Xanthomonas, a plant pathogen, and comprise translocation activation domain (AD), DNA binding domain, nuclear localization signals (NLS) (Figure 1B). The DNA binding domain determines the specificity of TALENs (27, 28). The Nuclease domain of TALENs refers to FokI. The amino-acid repeat sequences of the TALEs are located in the central position of the DNA binding domain and usually consists of 34 amino acids (29). However, variant repeats with 33 or 35 amino acids are not uncommon, and the last repeat of this domain is truncated at the 20th amino acids. Most TALEs have 5–30 repeats, with an average of 17 repeats. Polymorphism among repeats depends on amino-acid residue 12 and 13 called “repeat variable diresidue” (RVD). The last repeat contains only 20 amino acid residues and is often referred to as “half repeat.” There are more than 20 combinations of RVDs, and the most common ones are “HD,” “NG,” “HG,” “NN,” “NS,” “NI,” and “N*.” “N*” refers to the repeating domain formed by 33 residues (RVD loses one residue). RVD corresponds to the nucleotide in a one-to-one relationship. For instance, HD, NG, Ni, and NN correspond to nucleotides C, T, A, and G.

The first synthetic TAL effector, dHAX3, consists of 11 canonical repeats and a half repeat (533 residues in total) (30). A naturally occurring Tal effector, PthXo1, contains two cryptic repeats at the N terminal of the canonical repeat domains, ordered 0 and −1. The −1 cryptic repeat domain has no RVD connection to the T0 base on the DNA strand (the T0 base is highly conserved at the TAL recognition site and is required to activate the TAL effector) (30). Each TAL repeat forms two left-handed helix bundles, and the RVD is located at the junction of the two helix bundles (31). Each TAL effector is connected to the DNA chain at the 13th amino acid residue, while the 12th amino acid residue mainly plays an auxiliary role in structure, forming a hydrogen bond with the backbone carbonyl oxygen of the 8th residue. “HD” and “NN” have the strongest binding force, and “NI” has a weak effect.

The most frequently used TALENs compromise TALEs which locate target site and FokI nuclease which is responsible for cleavage of target site. As FokI nuclease requires homodimerization for activate cleavage, a pair of TALENs were designed to locate at the upstream and downstream sequences of the target site (32). Besides nucleases, TALEs could connect to transcription activators for promoting transcriptional processes (33); connect to transcription repressors for suppressing gene expression (34); connect to recombinases for modifying and recombining DNA (35). Although CRISPR has received more attention in recent years, and most of the published animal experiments of gene-editing therapy have used CRISPR for genetic modification, TALENs still have unique advantages in clinical applications. Compared to CRISPR, though TALENs editing is characterized as lower efficiency, the benefit inducing less off-target editing and minimal cytotoxicity overwhelms CRISPR in human gene-editing (36).

### CRISPR/Cas9

In 1987, Ishino et al. found a highly ordered and repeat DNA sequence in the IAP gene of *Escherichia coli* (37). In 2002, Jansen et al. analyzed and studied the sequence by *in silico* analysis. The sequence were called as clustered regularly interspaced short palindromic repeats (CRISPR) according to the unique DNA structure (38). They named the gene that adjacent to and functionally associated with CRISPR as Cas (CRISPR-associated), and found a total of 4 Cas genes (Cas1, Cas2, Cas3, Cas4) (39). In June 2012, Jinek et al. firstly demonstrated that CRISPR/Cas9 cloud cleave any DNA strand *in vitro*, pointed out the ability of CRISPR to modify genes in living cells, and thoroughly discussed the feasibility of CRISPR in genome editing (40). In January 2013, Long et al. achieved CRISPR gene-editing in mammalian cells, confirming that CRISPR/Cas9 gene-editing technology could be successfully applied to human genome (41).

CRISPR is an adaptive immune defense system that exists in bacteria and archaea against viruses, plasmids, and foreign nucleic acids (39). When bacteria are invaded by viruses or plasmids, or faced the invasion of exogenous DNA from viruses or plasmids, the system can capture the DNA sequence and store it in its spacer. Sequences in the spacer are then transcribed and further spliced and modified by CAS proteins to form crRNA (CRISPR RNA) that recognize the DNA of the invader (42). crRNA bridges trans activating RNA (tracrRNA) and Cas9 nuclease to form Cas9 complex (Figure 1C). The complex scans the entire exogenous DNA sequence and identifies the region of the invader genome sequence that is complementary to crRNA sequence. Although DSB activated DNA repair mechanism, the process is error-prone and insertions and deletions could occur, resulting in genetic loss-of-function and the availability of specific gene-knockout (43).

According to the new classification method proposed in 2020, CRISPR-Cas associated systems (CASSs) have been classified into two broad categories. Class1 CASSs compromise type I, type IV, and type III, and 33 subtypes. The new Class2 CASSs compromises type II and type V, and the newly classified type VI, with 17 subtypes in total (44).

Artificially engineered CRISPR/cas9 gene-editing system act through two components: Cas9 and single-guide RNA (sgRNA, comprising crRNA and tracrRNA). The first 20 nucleotides of the crRNA could be artificially synthesized to bind with the target sequence specially (45). Then Cas9 nuclease is guided by sgRNA to the target locus depending on the design of the first 20 nucleotides (46). By identifying the target gene PAM sequence of NGG (N: A, T, C, G), Cas9 cleaves both of the DNA strand to form DSB at 3–4 bases upstream of the PAM sequence. There are two primary mechanisms involved in DSB repair: non-homologous end-joining (NHEJ) and homology-directed repair (HDR) (47). NHEJ predominate the repair pathway when no repair template is provided. NHEJ is an error-prone DSB repair mechanism that triggers the unpredictable base fragments insertions, deletions or substitutions at the DSB site, causing desirable loss-of-function of target gene. Alternatively, if homologous donor templates are abundant, DSB will be predominantly repaired by HDR pathway. HDR could repair more precisely and generate knock-in, deletion, correction, and mutation of target genes.

Compared with the 2nd-generation (ZFNs and TALENs) gene-editing technology, the 3rd-generation CRISPR/Cas9 relies on RNA-DNA interaction rather than protein-DNA interaction for target site recognition, dramatically improving gene-editing accuracy. Another advantage is no necessary to modify the DNA recognition domain according to different DNA targets through the tedious process of protein engineering; instead, just need to synthesize 20 nucleotides. Therefore, CRISPR/Cas9 appears to be more convenient for large-scale gene-editing and is becoming more widely used in scientific research (48).

CRISPR/Cas9 genome editing technology has been applied in gene therapy of great potential, including editing, regulating, and monitoring individual genes at the genomic and epigenomic levels. In 2013, Wu et al. firstly demonstrated that the CRISPR/Cas9 system can directly correct genetic defects through NHEJ or HDR-mediated gene-editing in a mouse model (49). In 2014, Long et al. used CRISPR/Cas9gene-editing technology to rescue muscle weakness and shortened life-span in mouse model of Duchenne muscular dystrophy (DMD), an inherited X-linked disease characterized by severe muscular dystrophy (41). CRISPR/Cas9's excellent performance in pre-clinical studies has shown its great potential for treating genetic diseases in humans.

### Base Editor (BE)

Nucleases-mediated gene-editing technology such as CRISPR-Cas9 and TALENs generates double-strand DNA breaks (DSBs) and then inactivates genes by causing insertions and deletions (indels) at target sites. However, nucleases are associated with unpredictable outcomes like complex mixtures of products, gene translocations, and random off-target editing. Moreover, nucleases-mediated gene-editing technology is powerless against congenital diseases caused by point mutations, insertions, and deletions.

In 2016, the first-generation of cytosine base editor (CBE1) was developed by Liu et al. (50). The novel genome editing technology allows one base to be precisely substituted by another base with no dsDNA breaks and homologous templates are required. The CBE1 system originates from CRISPR/Cas9, and it comprises catalytically-dead Cas9 (dCas9) which was deactivated by Asp10Ala and His840Ala mutations, so the system only recognizes the targeting site but does not cleave the DNA strand. The N terminal of dCas9 was connected to a cytidine deaminase (e.g., hAID, hAPOBEC3G, rAPOBEC1, and pmCDA1) which functions as deaminating cytosine to uracil (Figure 1D). As a result, the uracil will be replaced by thymine and the paired guanine will be replaced by adenine after DNA replication. Nevertheless, the editing efficiency of the 1st generation BE in human cells is as low as 0.8–7.7%. The authors ascribed the disappointing base editing efficiency to Uracil DNA glycosylase (UDG), which eliminates Uracil from DNA and reverses the U-G pair to the original C-G pair. Since the UDG competes with the 1st generation BE, David R. L exploited the 2nd-generation base editor (BE2) by connecting Uracil DNA glycosylase inhibitor (UGI) to the C-terminus of BE1, achieving a total editing efficacy of 20% in human cells. Meanwhile, in the 3rd-generation base editor (BE3), dCas9 was substituted by nCas9(D10A) to nick non-edited strand, thereby initiating mismatch repair (MMR) of non-edited strand. In this process, edited strand (incorporating U) served as a template and U-A pairs were more produced and more T-A pairs were generated in next-step DNA replication. With this approach, editing efficiency climbed to 37% in human cells.

Following cytosine base editor (CBE), adenine base editor (ABE) was quickly exploited to convert A-T pairs to G-C pairs (51). The conversion process is that, adenosine was hydrolytically deaminated by adenine deaminase to generate inosine firstly, and then inosine will be read and replicated as guanine at DNA level. Therefore, A-T pairs were substituted by G-C pairs. The conversion process is efficient and secure in human cells (editing ~50% DNA and producing 0.1% indels in total).

However, concerns of off-target effects were raised for CBE in a mouse embryo editing experiment (52). Among various upgrades which have been made to address the substantial off-target effect (53–55), two of the most grateful upgrades are prime editor (PE) and dual BE. Zhang et al. (56) and Grunewald et al. (57) simultaneously developed dual-deaminase CRISPR base editor that could concurrently convert A and C to G and T by fusing both cytosine deaminase and adenine deaminase with Cas9 nickase. Dual BE demonstrated comparable editing efficiency and off-target effect with single BE.

PE is not only an innovative update from conventional BE, but superior to CRISPR/Cas9 because DNA repair is mediated by single-strand break rather than DSB and no template is required (58). Basic CBE and ABE could achieve four kind base conversions (C → T, G → A, A → G, and T → C), while the prime editing technology not only achieves all 12 kind base conversions, but also induce targeted insertions and deletions. Prime editing system comprises (i) prime editing guide RNA (pegRNA): including primer binding site (PBS) and reverses transcription template; (ii) reverse transcriptase (RT); (iii) nCas9 nickase (fused to RT) (58). Firstly, the pegRNA guided the fusion protein to the targeted locus, then nCas9 cut the single DNA strand (the one not complementary to pegRNA). In next step, pegRNA's PBS binds to the primer at cleavage site, and soon afterward, pegRNA's template was reverse transcribed to DNA strand, achieving bases substitution, insertions, and deletions. Although achieving more efficient editing, more severe indels were generated than BE because PE still causes single strand break.

Application of BE in congenital diseases caused by point mutations is now under pre-clinical investigation. In mouse sickle cell disease (SCD) model, Adenine base editor (ABE8e-NRCH) demonstrated the ability of ameliorating sickling morphological characteristic of reticulocytes, and the ability of converting pathogenic hemoglobin subunit beta (HBB) to benign HBB (59). Koblan et al. (60) recently reported their research using ABE to correct mutation (c.1824 C>T, G608G) in Lamin A/C gene (LMNA) which causes Hutchinson-Gilford progeria syndrome (HGPS). The correction efficiency is 90% at cell level and 20–60% at mouse level. Mutation correction by ABE successfully conserved vascular smooth muscle cell counts and ameliorated adventitial fibrosis.

## Gene Therapy and Cardiovascular Disease

### Gene Therapy in Atherosclerosis

Gene-editing technology has been used to uncover the specific role of genes in disease pathophysiology and biological mechanisms, and as a tool for disease prevention and treatment. Monogenic diseases and catastrophic diseases could be the targets of gene therapy (61–63). In the cardiovascular field, Gene-editing tools have already been applied in fundamental research investigating the mechanism of cardiovascular disease especially atherosclerosis (64). This opens the door to gene therapy for the cardiovascular system.

Atherosclerosis is a multifactorial systemic vascular disease that involves local immune-inflammatory processes in artery wall, triggering artery stenosis or occlusion in the middle and late stages (65, 66). In addition, rupture of unstable atherosclerotic plaque will form acute thrombosis because the exposure of extracellular matrix and smooth muscle cells triggers adhesion and activation of platelets and the coagulation cascade system (67). Ischemia of vital organs is the severe consequence of atherosclerosis, such as ischemic stroke, coronary artery disease, myocardial infarction, and lower limb ischemia. The 2019 ACC/AHA guideline recommends patients with severe atherosclerosis regularly take antiplatelet agents for secondary prevention, and patients with an intermediate or higher risk of atherosclerotic cardiovascular disease (ASCVD) should receive statin therapy (68). However, long-term intake of statins and antiplatelet agents leads to liver injury and high bleeding risk, respectively. These defects of traditional medical therapy highlight the transformation to new therapeutic strategies like gene therapy.

The pathogenic mechanism of atherosclerosis is complex because multiple factors like gene mutation, lifestyle habits, and environmental factors are involved in the pathogenesis (69). At present, the design of gene therapy for AS mainly targets its risk factors (e.g., hyperlipidemia, hypertension, diabetes). If the risk factors of atherosclerosis can be controlled at the genetic level, it will revolutionize the medical therapy era to the gene therapy era. Lipid deposition process the early occurrence and development of atherosclerosis. Hyperlipidemia, especially low-density lipoprotein cholesterol (LDL-C), has been shown to be most essential triggers of atherosclerosis pathogenesis and the independent risk factor of cardiovascular events (70).

#### Gene Therapy Targeting Lipid Metabolism

Naturally occurring loss of function mutations in pro-atherosclerotic genes have a protective effect on atherosclerotic vascular disease (64, 71, 72), and even in a homozygous or compound heterozygous state, where the gene is completely knocked out, there are no serious adverse health consequences. Three of the most well-known pro-atherosclerotic genes are proprotein convertase subtilisin/kexin type 9 (PCSK9) (73–75), angiopoietin-like protein 3 (ANGPTL3) (76–78), and Apolipoprotein C-III (ApoC3) (79–82), and they are all lipid metabolism related genes.

PCSK9 is a protein predominantly expressed in liver. Its function was unknown until 2003, the researchers identified a mutant PCSK9 gene in a French family gathered autosomal dominant hypercholesterolemia (83), thereby reputed PCSK9 as the third gene in addition to low-density lipoprotein receptor (LDLR) and ApoB associated with autosomal dominant familial hypercholesterolemia. PCSK9 protein combines with LDLR-LDL to form PCSK9-LDLR-LDL complex and transfer the complex to lysosomes for degradation, this process effectively prevents LDLR recycling to cell membrane. Gain-of-function mutation of PCSK9 increases the affinity for LDLR and accelerating its degradation, triggering autosomal dominant hypercholesterolemia and accelerating atherosclerosis progression (84–86). Intentional disrupting PCSK9 activity by loss of function mutations (87), therapeutic PCSK9 protein antibodies, or siRNA mediated gene silencing (88), could significantly reduce circulating LDL-C levels, inhibit cardiomyocytes autophagy, and lower the risk of coronary heart disease. Therefore, PCSK9 has become one of the most concerned and promising targets of atherosclerosis gene therapy. The monoclonal antibody targeted PCSK9 has been investigated in ODYSSEY trial (alirocumab) (89), FOURIER trial (evolocumab) (90) and SPIRE trials (bococizumab) (91). All indicated that PCSK9 reduced plasma LDL-C level by ~60% and major cardiovascular events. Meanwhile no security concerns were pointed out.

CRISPR technology is another promising way to knock down PCSK9 levels in the human liver once for all. Kiran's team from Harvard University has been being committed to this work (69, 92). The team firstly attempt to investigate the *in vivo* loss-function editing of PCSK9 in mice liver by ADV-CRISPR-Cas9 system (92). They observed that more than 50% of PCSK9 gene had loss-of-function mutation. However, no off-target mutagenesis occurred, and this led to reduction of PCSK9 protein level, uptrend of plasma LDLR level, and 35–40% reduction of total plasma cholesterol level. Soon afterward, the team transplanted human hepatocytes to FRG KO mice (Immune deficient mice) fed by NTBC, which killed endogenous mouse hepatocytes. Consequently, human hepatocytes dominated mouse liver function than native mouse hepatocytes. Then authors developed adenoviruses-CRISPR/SpCas9 system to target human PCSK9 gene (69). The loss-of-function mutation rates were 42–47%, indicating similar CRISPR/Cas9 delivery efficiency by adenovirus (ADV) and adeno-associated virus (AAV). Although total cholesterol levels did not decrease due to the compensatory expression of mouse PCSK9 protein, this study established the safety of direct genome editing human PCSK9 in human hepatocyte. Recently, Musunuru et al. published their latest research using CRISPR base editors to knock down PCSK9 in cynomolgus monkeys. The CRISPR base editors encouraged a significant reduction of plasma PCSK9 level (90%) and higher reduction of plasma LDL-C level (60%) (93).

Zhang Feng's lab developed AAV-SaCas9 system to disrupt PCSK9 in mouse liver, consequently reduced total cholesterol level (94). The authors also conducted genome editing in ApoB but observed lipid accumulation. Somatic disruption of ApoB leads to Liver micro-vesicular steatosis, which could cause endoplasmic reticulum stress because activated glucose regulated protein 78 (GRP78/BIP) and phosphorylated eukaryotic initiation factor 2 α (p-eIF2α) (27).

As another promising target for atherosclerosis, ApoC3 is predominantly expressed in liver and to a smaller extent in the intestine. After being secreted to plasma, it is situated in and forms a vital membrane component of high-density lipoproteins (HDL) and triglyceride-rich lipoproteins (TRL) including very-low-density lipoproteins (VLDL), intermediate-density lipoproteins (IDL), and chylomicrons. ApoC3 overexpression leads to progressive atherogenesis and post-surgery artery restenosis by accelerating SMC proliferation, and hyperlipidemia could be an additional factor (95). ApoC3 repress TRL uptaking by mediating LDLR; inhibits triglyceride (TG) degradation through inhibiting lipoprotein lipase (LPL) and hepatic lipase (HL) (96).

ApoC3 knockout hamster model was established by CRISPR/Cas9 system (97). As a result, when fed by a chow diet, triglyceride level was significantly reduced while total cholesterol and HDL-C were not. While fed by the western diet, triglyceride and total cholesterol levels were significantly reduced, and conversion of VLDL/LDL to HDL and a reduction of atherosclerotic lesion were observed. This study offered ApoC3 as a promising gene therapy target for hyperlipidemia-induced atherosclerosis.

Other potential targets for the treatment of atherosclerosis have also demonstrated promising efficiency in animal models. ApoA-I is the main protein constituent of HDL surface, and is responsible for reverse cholesterol transport (RCT) (98), helps accelerate cholesterol efflux, exerts anti-inflammatory and antioxidant effects (99). On the contrary, studies have shown that HDL with poor ApoA-I but high serum amyloid A (SAA), ceruloplasmin, and ApoC3 exhibits a pro-inflammatory and prooxidant effect, thus called them dysfunctional HDL (100, 101). Wacker et al. transferred Apolipoprotein A-I to the rabbit atherosclerosis model through adenoviral vector, observing decreased plaque volume and repressed inflammation (102).

Peroxisome proliferators-activated receptor γ - Liver X receptor α (PPARγ-LXRα) acts as a key regulator facilitating cholesterol efflux from macrophages to plasma through activating ATP-binding cassette transporter 1 (ABCA1)/ATP-binding cassette transporter G1 (ABCG1) (103, 104). Cholesterol efflux, the most vital step in reverse cholesterol transport, helps prevent the formation of macrophage foam cells, thereby reversing atherosclerotic lesions. Meanwhile, PPARγ in liver tissue promotes adipocytes differentiation and cholesterol accumulation in the liver, which lowers circulating cholesterol levels (98).

Hu et al. demonstrated that overexpression of PPARγ stabilized atherosclerotic plaques through reducing lipids deposition, alleviating macrophages infiltration, and smooth muscle cell proliferation (105). Transferring secretoneurin rescued blood flow, amputation rate, and vessel density in atherosclerosis-induced hind limb ischemia model, but no change in plaque area (106).

#### Gene Therapy Targeting Immunoreaction and Inflammation

Atherosclerosis is considered a chronic inflammatory disease. That is to say, pro-inflammatory factors favor the development of atherosclerotic plaque. As early as 2002, inhibiting monocyte chemoattractant protein-1 (MCP-1) in DNA level indicated the ability to reduce inflammatory cell infiltration, thereby stabilizing atherosclerotic plaque (107). Liver X receptors (LXRs), oxysterol-responsive transcription factors that regulate cholesterol metabolism, are potential targets for eliminating inflammatory responses in atherosclerosis (108). Li et al. transfected hematopoietic stem cells (HSCs) with lentivectors-loaded LXRα, then transplanted HSCs into LDLR^−/−^ mice atherosclerosis model. Both reduction of plasma triglyceride levels, and reduction of atherosclerotic plaque volume were observed. The protective effect on atherosclerosis is attributed to the enhanced expression of cholesterol efflux genes ABCA1 and apoE by LXRα. In addition, as LXRs are involved in the regulation of cytokine production [e.g., interleukin 1 beta (IL-1β), interleukin 6 (IL-6), and tumor necrosis factor-α (TNF-α)], IL-6 and TNF-α levels were down-regulated when LXRα was enhanced by transgene. Therefore, the authors suggest that transgenic LXRα involves inhibiting the progression of atherosclerosis by regulating both lipid metabolism and inflammatory response (109).

Immunoreaction and Inflammation play a vital role in atherosclerotic plaque progress. Chemokines involving recruitment of monocytes (e.g., E-selectin, P-selectin, ICAM-1, VCAM-1), inflammatory cytokines, and adaptive immunity (antigen-presenting cell, T cells, B cells were involved) could be potential target for gene therapy. But it remains unclear whether this affects systemic defense response. The focus of this study is that circumscribing gene therapy in atherosclerosis region without compromising tumor surveillance or function in immune defense.

#### Gene Therapy Targeting Non-coding RNA

Long non-coding RNAs (lncRNAs) are a class of RNAs coded more than 200 nucleotides without the ability to translate protein (110). Consensus has been made that LncRNAs are involved in the formation of atherosclerotic plaques and LncRNAs are potential targets for therapeutic intervention (111–113). lncRNA liver-expressed liver X receptor-induced sequence (LeXis) regulates lipid metabolism through mediating liver X receptors (LXRs) and RALY (a transcriptional cofactor for cholesterol biosynthetic genes) (114). Tontonoz et al. attempted to treat atherosclerosis caused by familial hypercholesterolemia by transfection of LeXis with AAV8. The successful transfection of LeXis down-regulated the expression levels of Srebp2, Fdps, Cyp51, Sqle, Hmgcr, and Fdft1 in the liver which were responsible for lipid metabolism. Cholesterol and triglyceride levels were down-regulated and atherosclerosis invasion area was reduced. At the same time, pathological sections showed its protective effect on fatty liver and no sign of hepatotoxicity was observed (115).

**Figure 2:**
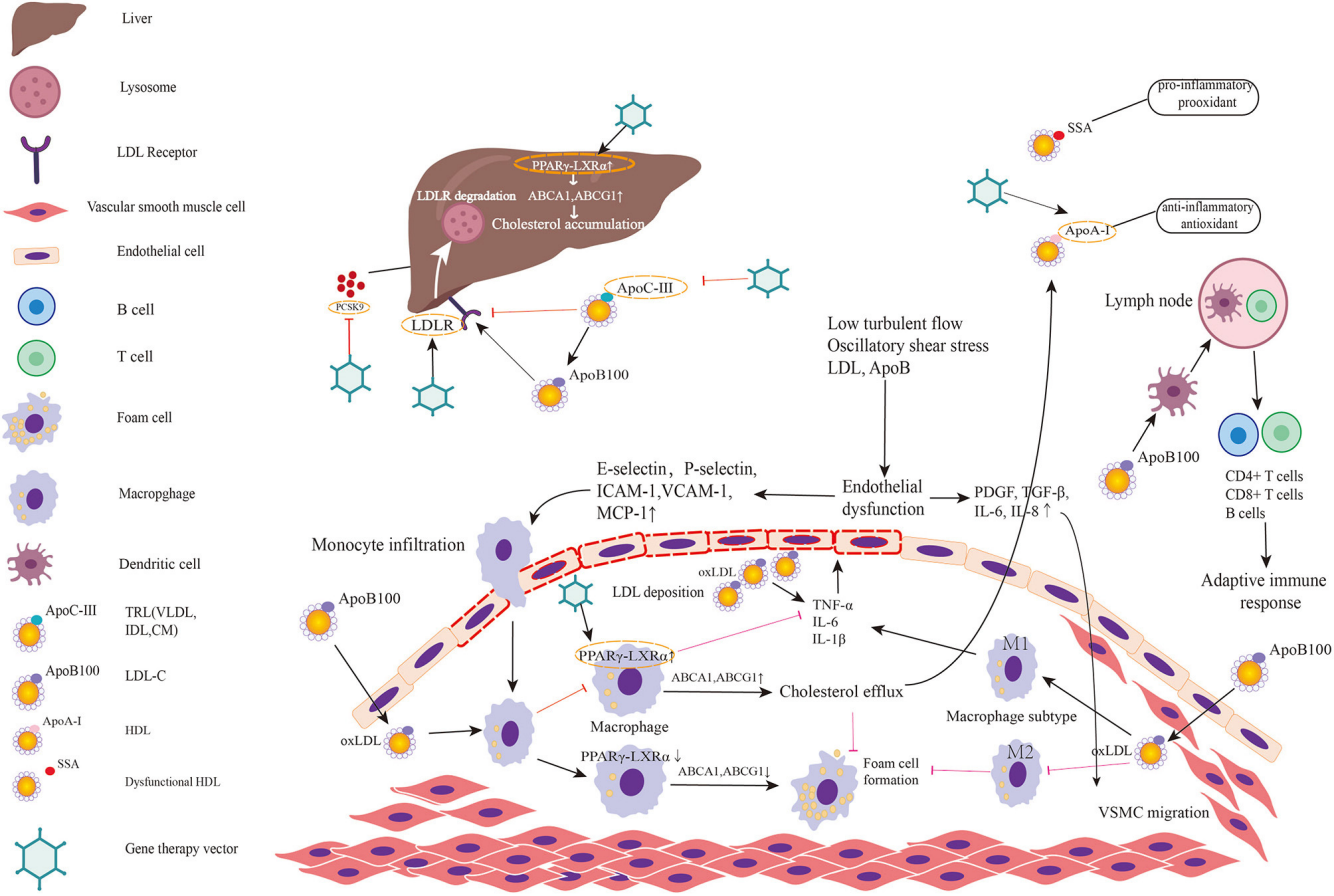
Targets for gene therapy of atherosclerosis and involved mechanism. The figure predominantly demonstrates two primary mechanisms of atherosclerosis: (1) lipid metabolism; (2) immunoreaction and inflammation. Low turbulent flow, oscillatory shear stress, LDL, and ApoB in plasma induce endothelial dysfunction and activate the inflammatory response in endothelial cells. Then endothelial inflammation triggers the expression of leukocyte adhesion molecules such as E and P-selectin, intercellular adhesion molecule-1 (ICAM-1), vascular cell adhesion molecule-1 (VCAM-1) on endothelial cell surface. These molecules promote monocytes adhered to endothelial cells and infiltrated into subendothelial layer. Monocyte chemotactic protein-1 (MCP-1) also helps recruit circulating monocytes to migrate to intima. Thereafter, monocytes differentiate into macrophages and engulf the oxidative LDL. The excessive accumulation of oxLDL in macrophages leads to foam cell formation. When PPARγ-LXRα in macrophage is upregulated, the overexpressed ABCA1 and ABCG1 promote cholesterol efflux to HDL, which is helpful to inhibit foam cell formation and prevent plaque progression. However, in pathologic conditions, PPARγ-LXRα is down-regulated thereby promoting foam cell formation. Meanwhile, endothelial dysfunction leads to secretion of PDGF, TGF-β, IL-6, IL-8 initiating VSMC migration. Among two major subtypes of macrophage, M1 predominates in athersclerotic plaque and secrets inflammatory factors while M2 exerts anti-inflammatory effects and anti-atherogenic effects. Antigen-presenting cells present LDL and ApoB to T cells in lymph nodes to activate adaptive immune response. Lipid metabolism involved mechanism has been elucidated in the manuscript. Targets in existing gene therapy experiments investigating atherosclerosis are marked by gene therapy vectors. In summary, gene therapies inactivating/disrupting PCSK9, ApoC3, and ApoB and gene therapies activating/correcting LDLR, ApoA-I, and PPARγ-LXRα play a protective role in atherosclerosis.

Non-coding RNA provides completely new targets for atherosclerosis but are not suitable for gene-editing therapy since no mutation occurs. In regarding to gene-replacement therapy, direct transfection of non-coding RNA may trigger unknown biological effect since its extensive binding sites of downstream target genes. The mechanism and targets of gene therapy for atherosclerosis were summarized in Figure 2, and related experiments are summarized in Table 2.

**Table 2:** Experiments of gene therapy for atherosclerosis.

**References**	**Gene editing tool (Vector)**	**Disease (Target)**	**Animal model**	**Efficiency**	**Security**
Ding et al. (92)	Crispr/Cas9 (ADV)	AS (PCSK9)	C57BL/6 mice	Mutagenesis rate of PCSK9: (>50%) Increased hepatic LDLR levels, Decreased plasma cholesterol levels	No off-target mutagenesis was detected
Wang et al. (69)	Crispr/SpCas9 (ADV)	AS (PCSK9)	FRG KO Mouse with human hepatocytes	Mutagenesis rate of PCSK9: 42–47% Total cholesterol levels not changed.	No off-target mutagenesis was detected
Musunuru et al. (93)	CRISPR Adenine base editor (lipid nanoparticles)	AS (PCSK9)	Macaca fascicularis	Mean base editing frequency: 63% Decreased LDL level: 90% Decreased plasma cholesterol level: 60%	One off-target editing site was detected (<1%) at the dose of 1.0 mg kg/L
Ran et al. (94)	Crispr/SaCas9 (AAV)	AS (PCSK9)	ApoB knockdown mouse	Mutagenesis rate of PCSK9: (>40%) Decreased total cholesterol levels: 40%	No off-target mutagenesis was detected
Guo et al. (97)	CRISPR/Cas9 (vector is unknown)	AS (ApoC3)	Hamsters	Decreased triglyceride and total cholesterol; Decreased atherosclerotic lesion; Conversion of VLDL/LDL to HDL;	Not report
Wacker et al. (102)	No gene editing (HDAd)	AS (ApoA1)	Rabbits	Decreased total cholesterol levels: 70% Decreased atherosclerotic lesion area Decreased Macrophage: 30%	Not report
Hu et al. (105)	No gene editing (ADV)	AS (PPARγ)	ApoE^−/−^ mice	Increased HDL level: 15.8% Decreased blood glucose level: 16.8% Decreased atherosclerotic lesion area Stabilized atherosclerotic plaque	Not report
Inoue et al. (107)	No gene editing (Plasmid)	AS (MCP-1)	ApoE^−/−^ mice	Decreased atherosclerotic lesion area Stabilized atherosclerotic plaque Decreased Immune cell infiltration	Not report
Li et al. (109)	No gene editing (Lentivectors)	AS (LXRα)	LDLR^−/−^ mice	Enhancing LXRα leads to: Decreased triglyceride: 50%; Decreased pro-inflammatory cytokines. Decreased atherosclerotic lesion area: 30%;	Not report
Tontonoz et al. (113)	No gene editing (AAV8)	AS (LeXis)	LDLR^−/−^ mice	Enhancing Lexis leads to: Decreased triglyceride and cholesterol Decreased atherosclerotic lesion area Protection of fatty liver	No sign of hepatotoxicity was observed

*HDAd, helper-dependent adenoviral vector; AS, atherosclerosis; AAV, adeno-associated virus; ADV, adenovirus; LDL, low-density lipoprotein; LDLR, low-density lipoprotein receptor; HDL, high-density lipoprotein*.

### Gene Therapy and Inherited Cardiovascular Diseases

#### Marfan Syndrome (MFS) and Other Syndromes

MFS is a connective tissue disease with autosomal dominant inheritance, originate from mutations of FBN1 encoding fibrillin-1. Fibrillin-1, a sizeable structural glycoprotein, exists in the extracellular matrix (ECM) and participates in the formation of microfibers that maintain the synthesis and homeostasis of elastic fibers in the aorta. Loss-of-function mutation of FBN1 leads to the thinning, fracture, reduced tensile strength and elastic recoil of the elastic fibers of the aorta in MFS patients, amplifying the likelihood of aortic aneurysms/dissections (116). In addition, mutated fibrinogen 1 lost the ability to bind to the latent TGF -β 1-binding protein (LTBP) to maintain TGF -β1 inactivity, making the transforming growth factor-beta (TGF -β) signaling pathway prone to be overactivated (117–119). Although TGF-β1 promotes matrix synthesis by activating the production of collagen and elastin, researches have shown that TGF-β1 could promote matrix degradation by increasing the production of plasminogen activators and stimulating the release of matrix metalloproteinase (MMP)−2 and MMP-9 from vascular smooth muscle cells (VSMCs) to ECM (120, 121).

The predominant clinical manifestations are skeletal, ocular, and cardiovascular involvement. Patients may develop mitral valve prolapse and aortic regurgitation, mainly in the Valsalva sinus, leading to aortic dissection and aortic rupture, and even death (116, 122). Zeng et al., for the first time, attempted genome modification of FBN1 mutations in MFS using a BE. An MFS patient was identified carrying heterozygous T7498C mutation of the FBN1 gene, which can be modified by the BE to achieve C to T conversion. The authors first attempted to establish and screen the homozygous FBN1T7498C cell mutation model, and then BE was located the T7498C mutation through the designed sgRNA and repaired the mutation. Results show that 8/20 colones implement correct repair (C to T conversion), but 2/20 mis-repair (C-to-g conversion) occurs. Subsequently, single sperms from MFS patients and immature oocytes from donors were fertilized *in vitro* to produce embryo model. mRNA of BE3 and sgRNA was injected into seven zygotes 16–18 h after *in vitro* fertilization, and seven zygotes were injected BE3 mRNA and scrambled sgRNA as control. Two days after, Sanger sequencing revealed all seven embryos achieved a near 100% correction at the 7498 site. Whereas, an unwanted C-to-T conversion occurred near the target site in one embryo (with a proportion of ~20%). To test security, seven edited embryos and three control embryos were tested for potential off-target sites by PCR, and four nt mismatches were observed. whole-genome sequencing of one corrected embryo and two control embryos reveals of off-target site. Corrected the pathogenic mutation FBN1 (T7498C) of Marfan syndrome in HEK293T cells and in heterozygous human embryos using the BE system, showing an overall correction rate of 89% and no detection of off-target, insertions/deletions (indels) in intended sites. The study suggested the superiority of BE over CRISPR/Cas9 treating MFS since it was DSB-independent and fewer off-target effects occurred (2).

For a better understanding of the pathogenesis of MFS and developing effective therapeutics, gene-editing technology was used for developing MFS models imitating the genetic pathogenesis of MFS. Borsoi et al. modified FBN1 gene in healthy donor induced pluripotent stem cells (hiPSCs) using CRISPR/Cas9 and demonstrated its pluripotency, three-germ differentiation potential, and genomic integrity (123). Due to the physiological and anatomical similarities between pigs and humans, Umeyama et al. constructed a heterozygous FBN1 mutant pig clone model by genome editing technology and somatic cell nuclear transfer technology, showing the gene defects in this specific genetic background and presenting complex disease phenotypes (124).

In addition to Marfan syndrome, other syndromes have phenotypes similar to MFS but with lower prevalence, such as Loys-Dietz syndrome (LDS) and Shprintzen-Goldberg Syndrome (SGS), etc. LDS and SGS showed considerable overlapping in clinical manifestation with Marfan syndrome. Compared with MFS, LDS is an autosomal dominant disease that develops earlier, progresses faster, has a wider range of aortic aneurysms. And LDS is more likely to involve the aortic arch, vertebral, and carotid arteries, as well as craniosynostosis, hyperopia, cleft/wide uvula or cleft palate, a tendency to severe allergies, and intestinal disorders (125). The mutated genes that cause LDS are basically part of the TGF-β signaling pathway, including TGFBR1, TGFBR2, SMAD3, TGFB2, TGFB3, and TGFB3. Heterozygous mutations in these key genes lead to partial loss-of-function of TGF-β signaling pathway (126–130). However, overexpression of TGF-β signaling can be detected in the aortic tissue of patients with LDS, which is caused by overcompensation of non-classical pathways, and the exact role of TGF-β signaling in the progression of aneurysms remains controversial (129).

In order to better investigate the mechanism of SMAD3 mutation in patients with aortic root aneurysms, Gong et al., using CRISPR-Cas9 technology, introduced a shifts mutation and nonsense-mediated decay of SMAD3 into Human pluripotent stem cells (hPSCs) to construct an LDS model (131). In addition to the characteristic features of MFS (involvement of the skeletal, ocular, and cardiovascular systems), patients with SGS also present with severe skeletal muscle dystonia and delayed mental development (126). Mutation of SKI proto-oncogene (SKI) is believed to be the trigger of the SGS. SKI is a transcriptional suppressor that inhibits TGF-β signaling pathway. However, as for the role of TGF-β signaling pathway in the pathogenesis of SGS (is it caused by increased or decreased TGF-β signaling pathway), there is still disagreement among scholars. One view is that when SKI is mutated, it leads to over-activation of TGF-β signaling pathway, which is consistent with the pathogenesis of MFS (126). Another point of view is that the transcriptional co-repressor encoded by SKI can participate in blocking the transmission of TGF-β signaling pathway, and the former is rapidly degraded under the mediation of ligand; When SKI gene mutations occur, the transcription suppressor becomes stable and thus resistant to ligand-induced SKI degradation, resulting in decreased expression of TGF-β signaling pathway (132).

In conclusion, dysfunction of TGF-β signaling pathway plays a pathogenic role in LDS, and the detailed mechanism still needs further exploration and verification. At present, we have not found any basic research or clinical trial about gene therapy for LDS and SGS, and it may be ascribed to the unknown pathogenesis too much mutated genes. Further studies should focus on identifying core pathogenic gene mutation and developing multiple gene editing technology.

#### Familial Thoracic Aortic Aneurysm and Dissection (FTAAD)

FTAAD refers to the occurrence of two or more thoracic aortic aneurysms/dissections in a family, except sporadic or syndrome-induced (such as Marfan syndrome, etc.), and the patients have no apparent abnormalities of other systems except aortic pathology (133). At present, loss-of-function mutation of ACTA2 encoding alpha-smooth muscle actin is considered to be the leading cause of familial TAAD. In addition, mutated genes that cause syndromes such as MFS (FBN1, TGFBR2, TGFB2, and TGFB3) can also trigger FTAAD (131). At present, no preclinical experiment using gene therapy for FTAAD is reported. As a polygenic disease, the lack of Experimental gene therapy is ascribed to the technical difficulty in establishing animal models of FTAAD. Moreover, technically difficult still exists to edit multiple loci simultaneously, therefore, the International Commission on the Clinical Use of Human Germline Genome Editing does not recommend heritable gene therapy for multigene disease currently (see Table 1).

#### Familial Hypercholesterolemia (FH)

FH is a common autosomal monogenic genetic disorder characterized by significantly elevated cholesterol and low-density lipoprotein cholesterol (LDC-C) levels, early xanthoma, and progressive ASCVD (3). The most common mutation occurs in the gene encoding LDLR, while other mutations were found in ApoB and PCSK9 (134). All of the mutations were inherited in an autosomal dominant way.

Experimental attempts have been made to investigate the potential of gene-editing in the treatment of FH caused by LDLR gene mutation. In 1995, Grossman et al. conducted the first human clinical trial of gene therapy in FH. Autologous hepatocytes were genetically engineered by recombinant retroviruses carrying the functional human LDLR, and then were transplanted into five patients' livers by portal vein infusion. Four months after infusion, 3 of 5 patients achieved a slight decrease in LDL-C levels (6–25%), total cholesterol (6–20%), and ApoB (10–21%). The trial failed because only a small number of hepatocytes expressing normal LDLR were detected in liver biopsies after 4 months of infusion and the degree of blood lipid reduction is not satisfactory. The result is likely attributed to the low *in vivo* transfection efficiency of retroviruses and it seems that functional LDLR genes were not integrated into the hepatocytes genome (135). We believe that the transfection efficacy could be improved by switching appropriate delivering system, which is discussed in section Delivering System in Gene Therapy.

Zhao et al. first used CRISPR/Cas9 system to produce a homozygous E208X mutant (GAG>TAG) in the fourth exon of LDLR gene through homologous directed repair (HDR) in mouse fertilized eggs, generating a complete LDLR loss-of-function mouse model (called LDLR^E208X^); an additional silent mutation in downstream (ATC > ATA) was introduced to prevent sgRNA binding and to recut the sequence (63). The LDLR^E208X^ mice exhibited exactly similar phenotypes and pathological changes like atherosclerotic lesions, lipid accumulation, SMC phenotype conversion, macrophages infiltration.

Subsequently, the authors used dual AAV8 system to transport Cas9 and sgRNA separately into neonatal hepatocytes (63). In the process, a liver-specific thyroxine-binding globulin promoter was connected to the Cas9 sequence for targeting liver tissue. Sanger sequencing revealed a 6.7% mutation correction rate, and off-target sites were observed but located in introns of several genes. The gene-editing therapy restored LDLR mRNA level (11% of wild-type) and restored LDLR protein level (18% of wild-type); meanwhile reduced atherosclerotic lesion area, lipid accumulation, macrophage infiltration, and plaque fibrosis. Both biochemical detection and histological staining did not show any sign of liver injury. This study shows great potential for gene-editing in liver cells to treat FH, but the unsatisfactory editing efficiency and undesired off-target effect should be addressed before clinical application.

The first-in-human study intended to evaluate the safety and effectiveness of AAV-transported LDLR gene therapy for Homozygous Familial Hypercholesterolemia (HoFH) was completed in Nov 2020 (NCT 02651675). The clinical trial involved nine participants without a control group; with the primary endpoint is adverse events and the secondary endpoints are percent change in lipid parameters such as LDL-C, total cholesterol (TC), non-HDL-C, HDL-C, TG, VLDL-C, lipoprotein(a), apolipoprotein B, and apolipoprotein A-I. Although the trial ended, the results have not been completed published. It is known that all patients were free from symptomatic adverse event, and, although they showed elevated transaminases, it could be rescued rapidly by steroid therapy. No dose-related toxicity was observed when the dose of AAV8 vector was 6.0 × 10^13^ gc/kg or less. At the dose of 7.5 × 10^12^ gc/kg, the serum cholesterol level of mice decreased by more than 80%. However, further increases in vector dose did not further reduce serum cholesterol levels (136).

In heterozygous LDLR mutation individuals, residual partial LDLR function exists (137). Coupled with appropriate treatment such as statins, LDLR can be maintained at an acceptable level. Whereas, homozygous LDLR mutation patients, due to almost no functional LDLR, exhibit no response to any medical therapy even PCSK9 inhibitors. The estimated efficacy of gene therapy for FH requires only partial restoration of LDLR levels, and combined drug-assisted therapy help significantly control the progression of atherosclerosis. Therefore, only a portion of liver cells needs to be genetically corrected, which can effectively reduce the probability of adverse events. Current animal experimental designs and clinical trials target homozygous FH. Once the safety of LDLR gene therapy is established, gene therapy for heterozygous FH or non-genetic AS patients could be attempted.

#### Heritable Pulmonary Arterial Hypertension (HPAH)

The characteristic pathological manifestations of HPAH are progressive vascular endothelial cell proliferation, smooth muscle cell hypertrophy, and adventitial thickening. These pathological changes lead to vascular remodeling and obstruction of precapillary pulmonary arteries, resulting in irreversible elevated pulmonary vascular resistance, right heart pressure overload, right heart failure, and even death (138, 139). Currently, loss-of-function mutations in bone morphogenetic protein receptor-2 (BMPR2), which encodes TGF-βII receptor, are considered the most vital pathogenic factor. This disrupts TGF-β pathway-related ligand binding, affects serine/threonine kinase activity, and leads to heterodimer formation (140). In addition, mutations in BMPR1B, ACVRIJ, ENG, SMAD9, CAV1, and KCNK3 genes were also shown to be closely related to HPAH. Fifty to eighty percent of HPAH patients carry mutations in BMPR2, with the penetrance of mutation in male carriers, which was about 14%, and that in female carriers was nearly 42% (141). Decreased BMPR2 expression and decreased BMP signaling due to heterozygous deletion mutations appear to be a common pathway in inherited PAH and idiopathic PAH.

Therapeutic strategies include replacing or enhancing defective BMP ligand signals, restoring or even enhancing BMPR2 signaling and preventing its degradation, inhibiting TGF-β signaling from maintain the balance between BMP and TGF-β, and modifying the downstream molecules of BMP/TGF-β signaling (142–147). The above strategies regulate the endothelial homeostasis or smooth muscle proliferation to ameliorate the symptoms of pulmonary hypertension and delay pulmonary hypertension progress. Reynolds et al. used angiotensin-converting enzyme (ACE) monoclonal antibodies to transfer BMPR2 gene packaging adenovirus to rat lung endothelial cells. The research conjugated AD with a bispecific antibody allowing AD target to the pulmonary artery since the antibody binds to the highly expressed angiotensin-converting enzyme on pulmonary endothelial cells. Chronic hypoxia and monocrotaline (MCT) rat models were established. Although BMPR2 gene expression was decreased in the two models, it could not completely simulate HAPH, thus the BMPR2 knockout model is a better solution. Hypoxia was evaluated after 3 weeks, MCT was evaluated after 10 days. Compared with untreated rats, adenovirus-transfected rats showed a significant reduction in pulmonary vascular resistance [total pulmonary vascular resistance (TPVR) decreased by 38% and pulmonary vascular resistance index (PVRI) decreased by 48%], a significant 40% reduction in vascular smooth muscle area per unit area of visual field, and a reduction in abnormally elevated TGF-β signaling by ~29% (145). However, more studies are needed to determine the safety and efficacy of editing these targets.

In Total, gene therapy for severe monogenic cardiovascular diseases is ethically feasible, and the therapeutic target could be clearly identified with DNA sequencing. The unconquerable limitation at present is the inefficiency of editing system and the security problem caused by off-target effect. The experiments of gene therapy for inherited cardiovascular diseases were summarized in Table 3.

**Table 3:** Experiments of gene therapy for hereditary cardiovascular diseases.

**Disease (Pathogenic gene)**	**References**	**Gene therapy method**	**Model**	**Efficiency**	**Security**
MFS (FBN1)	Zeng et al. (2)	Crispr-BE	homozygous FBN1^T7498C^ HEK293T Cells	40% of mutants were corrected	10% unpredicted base conversion occurred
			Heterozygous FBN1^T7498^ embryos	89% of mutants were corrected	1 of 7 embryo showed unpredicted base conversion
FH (LDLR, APOB, PCSK9)	Grossman et al. (135)	Hepatocytes transfected by retroviruses; Transfected Hepatocytes were infused to portal vein	Five patients with homozygous FH	3 of 5 patients decreased total cholesterol (6–20%) 3 of 5 patients decreased LDL (6–25%) 3 of 5 patients decreased ApoB (10–21%)	Two of five patients developed perioperative myocardial ischemia No immune response occurred against to LDLR and retrovirus
	Zhao et al. (63)	AAV8-CRISPR/Cas9	Ldlr^E208X^ mice	Restored Ldlr mRNA level (11% of wild-type) Restored LDLR protein level (18% of wild-type) Decreased atherosclerotic lesion area Alleviated lipid accumulation Decreased macrophage infiltration Decreased plaque fibrosis	Off-target sites were observed but located in introns of several genes No sign of liver injury detected.
HPAH (BMPR2)	Reynolds et al. (145)	ADV transgene	Chronic hypoxia rat model; Monocrotaline (MCT) rat model;	Reduction in pulmonary vascular resistance (TPVR:38% and PVRI: 48%), Reduction in vascular smooth muscle area per unit area of visual field (40%) Reduction in abnormally elevated TGF-β signaling by ~29%	

*MFS, marfan syndrome; FH, familial hypercholesterolemia; HPAH, heritable pulmonary artery hypertension*.

## Gene Therapy and in-Stent Restenosis

Artery severe stenosis or occlusion due to atherosclerosis can lead to acute or chronic ischemia of the myocardium, brain, and peripheral organs. At present, angioplasty and stent implantation have become one of the primary surgical treatment methods for arteriosclerotic obliterans (148). However, in-stent restenosis (ISR) and stent thrombosis (ST) are serious postoperative complications that raise extensive concerns. ISR could be diagnosed as the stenosis rate of stent lumen beyond 50% by angiography. The predominant cause of ISR is the hyperplasia of the new intima in stents (149). Prior to stent implantation, a balloon is usually used to predilate the narrow artery, which leads to local damage to arterial intima. In addition, local stimulation of stents can also cause chronic injury to arterial endothelial cells. Exposure of collagen and fibronectin caused by damage of endothelial cells triggers local platelets aggregation and activation, initiating coagulation cascades and leukocyte recruitment. Leukocytes–platelets interaction releases numerous cytokines and chemokines (150). Macrophages engulf cell fragments in tissues and secrete cytokines such as TNF-α, IL-6, TGF-β, and reactive oxygen species (ROS) (150). Subsequently, VSMCs proliferated and migrated to the intima under the action of platelet-derived growth factor (PDGF) A/B, TNF-α, IL-6, IL-8, TGF-β, ROS, and other cytokines. Partial VSMCs could be transformed from contractile phenotype to secretory phenotype, secreting proteic acids and proteoglycans, which further increase the extracellular matrix (EMC) and subsequently form new intima (151).

Compared with bare stents (BES), drug-eluting stents (DES) reduce platelet activation and aggregation, inhibit migration and proliferation of smooth muscle cells (SMCs) and endothelial cells (ECs), and significantly reduce re-stenosis rate and early stent thrombosis by slowly releasing drugs stored in the coating (152). However, drug-eluting stents still face the challenge of cytotoxicity and non-specific drug effect. And DES inhibits the process of re-endothelialization while inhibiting neointimal hyperplasia, which increases the risk of late stent thrombosis (153). As a result, patients need to take anticoagulant drugs for longer-term, amplifying the severity of the bleeding events for patients (154). In addition, from the perspective of long-term effects, DES seems to only delay the occurrence of ISR but does not fundamentally prevent late ISR (155). Moreover, neoatherosclerosis, which refers to the development of new atherosclerotic plaque in the stent after stent implantation, also contributes to ISR. Some studies have shown that the occurrence of neoatherosclerosis in the first-generation DES is even earlier than in BES (156).

**Figure 3:**
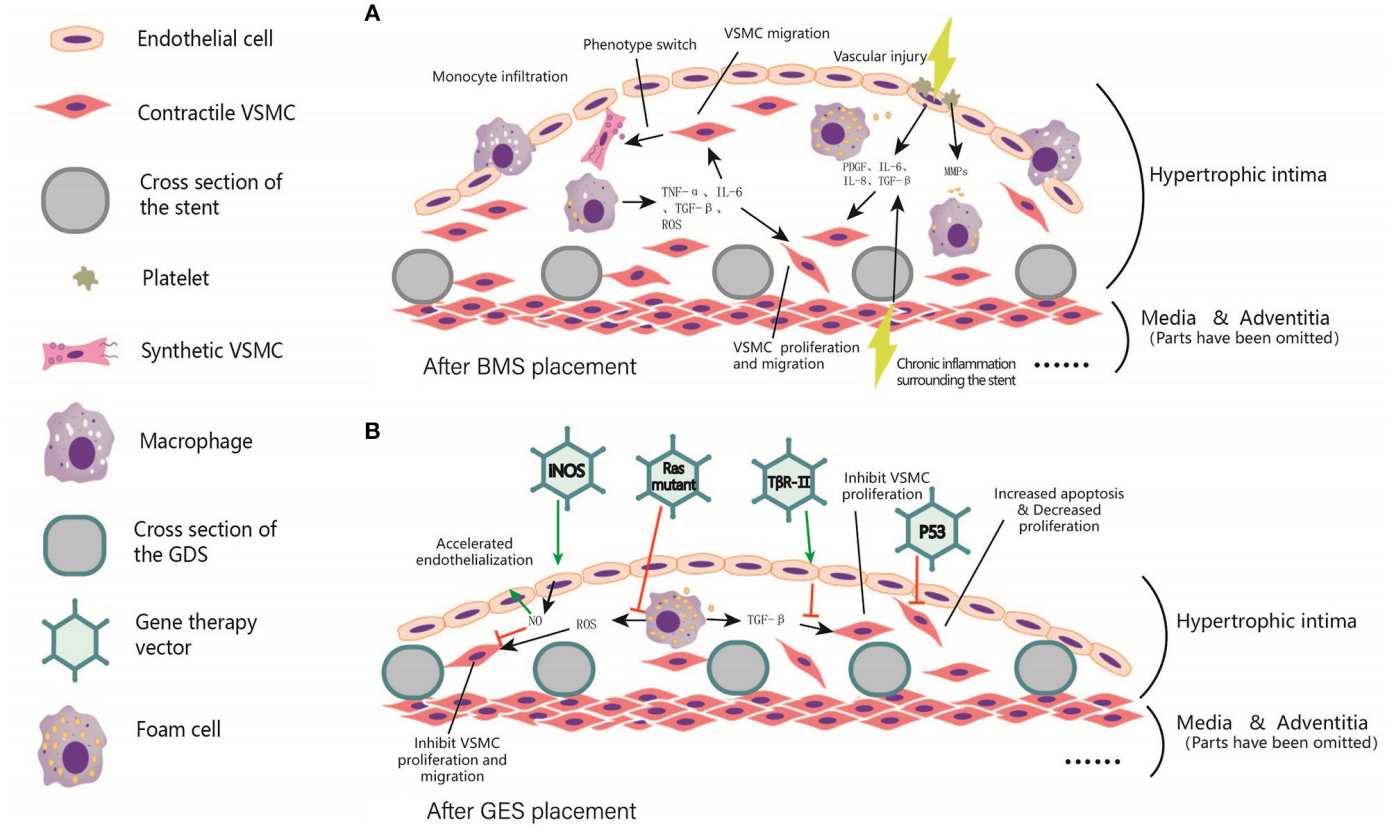
Local intimal hyperplasia in arterial lumen after BMS and GES implantation. **(A)** Before stent implantation, A balloon is usually used to predilate the stenosis segment of the artery, resulting in local damage to the intima. In addition, chronic inflammation occurs surrounding the stents. Subsequently, platelet aggregation, invasion of inflammatory cells, and cause the release of TNF-α, PDGF, IL-6, IL-8, TGF-β, ROS, and other inflammatory mediators were observed. These factors trigger VSMCs proliferation, and even foam cells formation. **(B)** After the coating was added to the scaffold skeleton, vectors were loaded to the coating for delivering the target gene to local endothelial cells, smooth muscle cells, fibroblasts and macrophages, etc. These target genes include iNOS, p53, Ras Mutant and TβR-II, etc. In general, the expression of target genes can inhibit the excessive proliferation of smooth muscle cells, reduce the generation of extracellular matrix, and accelerate endothelialization, thus reducing the process of in-stent restenosis and the occurrence of in-stent thrombosis.

An interesting solution is that gene eluting stents (GES) extend the elution time and allow longer stent patency through slow local gene modification. Local transferring of certain genes has been verified to be effective in inhibiting neointimal hyperplasia and neoatherosclerosis. These genes include Inducible NOS (iNOS) (157), vascular endothelial growth factor (VEGF) (158), tissue inhibitor of metalloproteinase 1 (TIMP-1) (159), monocyte chemoattractant protein-1(MCP-1) (160), Ras mutation (161), TGF-β1 receptor Type II (TβRII) (162). iNOS is rarely expressed under physiological conditions but highly expressed under inflammatory stimuli. The delivery of iNOS increases local NO content, which is conducive to the process of re-endothelialization and inhibits the further adhesion of platelets and mononuclear macrophages, thus inhibiting the proliferation and migration of VSMCs (157). The local high expression of VEGF benefits the regeneration of vascular endothelial cells, and further promotes re-endothelialization (163). Matrix metalloproteinases (MMPs) degrade the extracellular matrix and recruit white blood cells to release large amounts of cytokines and chemokines promoting proliferation and migration of VSMCs. MMPs activity was inhibited by tissue inhibitors of matrix metalloproteinases (TIMPs). Therefore, Ramirez Correa et al. used AAV to deliver TIMP1 gene into a rat model of carotid intimal hyperplasia and observed a 70.5% reduction of carotid intima thickness compared with model group after 2 weeks (159). Wild-type p53 (WT-p53) inactivates G1 cyclin by activating P21waf-1/Cip-1/SDI-1. Yonemitsu et al. transfected Japanese hemagglutinin virus/liposome carried WT-p53 gene into rabbit carotid artery after balloon injury. Results found inhibited VSMCs proliferation and neointima formation, which may be helpful to prevent ISR (164). The proto-oncogene C-H-RAS is considered to be closely related to cell growth and proliferation. After the mutated RAS gene is introduced into VSMCs by adenovirus, mutated proteins have a dominant role in VSMCs completely inhibiting the activation of mitogen-activated protein kinase and inhibiting DNA synthesis (161, 165). The mechanism and targets of gene therapy for in-stent restenosis were summarized in Figure 3.

In general, most of the target genes are transmitted to promote arterial re-endothelialization and reduce the proliferation and metastasis of VSMCs, thereby reducing intimal hyperplasia. Moreover, when the transferring a single gene cannot achieve the expected effect, the combined transferring of two or more genes may be a better solution. As a combination of gene therapy and endovascular therapy, gene eluting stents could function as a physical delivering system helping virus or liposomes to locate in specific arterial segments. Future studies on GES may focus on the following aspects: (1) Selection of more specific and efficient targets and search for gene delivery vectors matching stents; (2) The currently used organic polymer coating still causes acute/chronic inflammation of arterial endothelium, thus the coating with less cytotoxicity and higher security level needs development; (3) Current studies are limited to gene replacement therapy, local somatic gene-editing mediated by CRISPR/Cas9 system, and/or base editing technology may play a more efficient role in preventing stent restenosis and neoatherosclerosis.

## Delivering System in Gene Therapy

Delivering a designed gene-editing system to targeted tissue is a crucial step of the whole gene-editing process. Take CRISPR/Cas9 as an example, the system could be transferred as three forms: (1) DNA encoding Cas9 and sgRNA; (2) mRNA encoding Cas9 and sgRNA; (3) Cas9 protein and sgRNA. The unstable and anion-charged nature of nucleic acid poses an obstacle to the alone gene-editing system for passing through cell membrane. Therefore, delivering vectors are required to help encapsulate nucleic acid or other components, making it easy to cross the membrane and avoiding the destruction of nucleases and proteases.

### Viral Vectors

The most classic delivery system is viral vectors, such as AAV, ADV, and lentivirus (LV). AAV, the most promising viral vector for its high affinity to a specific tissue, introduces non-integrated gene transfection, but the primary limitation is low packaging capacity (166–168). Various AAV serotypes and AAV variants were found to target particular tissue (169). Unlike non-viral vehicles and physical delivering, AAV vehicles could guarantee a continued provision of DNA, since the ability to self-replicate AAV genome replication and AAV DNA fragments could be integrated into the host DNA. Given the diameter of AAV is ~20 nm and the maximum of AAV capacity is 4.5–5 kb, “all-in-one” packaging is a challenge for AAV. Researchers must separately package the SpCas9 and sgRNA into two AAVs and co-transfer them. Cas9 from *Staphylococcus aureus* (SaCas9) only take 70% size of SpCas9, not only achieving “all-in-one” packaging but also leaving free space for tags and markers (94). Although some groups tried ADV (92, 170, 171) or LV (172, 173) vectors to deliver CRISPR/Cas9 components *in vivo*, their strong immunogenicity prevented further *in vivo* transfection and clinical translation. LV is prone to mediate the integration of the transferred gene into the host genome, resulted in random insertion of genetic elements, activation of proto-oncogenes, and insertional mutagenesis which constitute the main causes of genotoxicity (174).

### Non-viral Vectors

Other non-viral delivery systems such as lipid nanoparticles/liposomes (175–177), polycations (178, 179), and other inorganic nanoparticles are also exploited to overcome the drawbacks of viral vectors. The advantages of non-viral vectors are the rare cellular toxicity, low immunogenicity, and no capacity limitation. The positively charged polycations parceled the negatively charged RNAs and DNAs and played a protective role until they were released in the intracellular matrix. liposomes are the most widely applied and investigated vectors due to their excellent biocompatibility and membrane affinity. The transfection efficiency depends on fusogenicity, size, surface charge, PEGylation, ligand modification. Cationic liposome vectors produced by monovalent or Multivalent cationic lipids are most widely used. Promoting transfection efficiency should overcome three barriers: long blood circulation time, vascular endothelium cells barrier, and efficient cellular uptake. For instance, PEGylation help liposomes live more stable and more prolonged in blood circulation by reducing intake by mononuclear phagocyte system. Modifying liposome surface ligand could help target cardiovascular components such as endothelial cells and vascular smooth muscle cells.

### Physical Delivery Methods

In addition, physical delivery methods such as microinjection and electroporation are suited for *in vitro* transfection. Microinjection physically overcomes the barriers of extracellular matrices, cell membranes, and cytoplasmic components by directly injecting components into cells using a 0.5–5.0 μm diameter needle under a microscope (180). Microinjection is reputed to be the ‘gold standard’ for delivering CRISPR system since (1) no limitation in cargo size; (2) 100 percent efficiency without affecting surrounding tissue/cells (181). Thus, microinjection is considered the most efficient method of introducing a gene-editing system into zygote cytoplasm for full-term mice with anticipated modification in all cells. Electroporation is commonly used in *in vitro* cells transfection. Electroporation involves applying pulsed high-voltage electrical currents to form a transient nanometer-sized pore in cell membrane, guaranteeing designed genetic components flow into the cell (182). Although the traditional view holds that electroporation is not suitable for *in vivo* delivery, recent researches enhance its *in vivo* suitability in muscle (183) and neuron (184).

### Choose Suitable Delivery System for Cardiovascular System

Due to the anatomical characteristics of the cardiovascular system, blood flow connects every organ or tissue with each other through systemic circulation, thus the vector requires high specificity and affinity for cardiovascular tissue. The vectors targeting cardiovascular tissue may travel with the circulatory system to any peripheral organs, especially reproductive organs. Therefore, possibility of vectors entering the sperm or ovum and resulting in HHGE cannot be ignored. VSMCs are major component of great arteries and the predominant target cell for cardiovascular gene-editing. However, the transfection efficiency of VSMCs was unsatisfactory (185, 186). Hydroxyl-rich Polycations have shown promising transfection efficiency, ability to resist circular protein aggregation, low cytotoxicity, and efficient cellular internalization in mice hearts (187). Zhang et al. exploited cholesterol-terminated ethanolamine-aminated poly (CHO-PGEA), which is Hydroxyl-rich and theoretically has high transfection efficiency and stability (188). This system with high packaging capacity exhibited excellent delivery efficiency to aorta tissue and no abnormal or inflammatory reaction occurred. Besides, the ideal vector should allow the editing system to be transported to specific arterial segments, as is applicable to FTAAD, HPAH, and MFS. The category of common delivery vectors or methods for gene therapy were summarized in Table 4. In conclusion, the ideal carrier suitable for cardiovascular disease should have the following characteristics: (1) high packaging capacity; (2) without cytotoxicity, genotoxicity, and immunogenicity; (3) high cardiovascular tissue specificity; (4) easy preparation and low cost; (5) high transferring efficiency.

**Table 4:** Common delivery vectors or methods for gene therapy.

**Categories**	**Vectors/methods**	**Advantages**	**Disadvantages**
Viral vectors	AAV	Efficient delivery; sustained expression; Low genotoxicity, immunogenicity; The most widely used viral vector in clinical application; Multiple serotypes for various tissue tropism	Low capacity (0~4.5 kb)
	ADV	High capacity (8–30 kb)	High immunogenicity
Non-viral vectors	Nanoparticles, nanoparticles, polycations	Unlimited packaging capacity Rare cytotoxicity, low immunogenicity Simple manipulation; Low cost	Relatively inefficient delivery
	Cationic liposome	The most widely used non-viral vector in clinical application; Excellent biocompatibility and membrane affinity	
Physical delivery methods	Microinjection	No limitation in cargo size; 100 percent efficiency; Low cytotoxicity; Suitable for gene therapy in zygote or single cell	Unsuitable for gene therapy in tissue, organs, or a large number of cells
	Electroporation	High delivery efficiency; Delivery to cell population	Unsuitable for *in vivo* gene therapy; Medium-high cytotoxicity

*AAV, adeno-associated virus; ADV, adenovirus*.

## Conclusion

The update from gene replacement therapy to gene editing therapy is a revolutionary development in gene therapy history. Gene editing not only provides an alternative to conventional cardiovascular diseases but is also the only potential cure for severe inherited cardiovascular diseases. Somatic gene-editing permanently solves the problem of insufficient production of functional molecules and excessive mutagenic products. At the same time, HHGE gains the potential to prevent the transmission of pathogenic variants to the next generation, which effectively reduces the proportion of pathogenic alleles in the total population. Currently, gene editing therapy is still in the pre-clinical stage, and the defects that limit its clinical application are low editing efficiency, obvious off-target effect, high toxicity and low tissue specificity of the delivery system, and insufficient recognition of the pathogenic genes of cardiovascular disease, etc. Once the technical issues are resolved and the balance between the advantages and disadvantages of gene therapy is tilted, the ethical limitations in diseases treatment are also expected to be reduced, but it should still be carried out under strict government monitoring. Of note, any gene-editing designed to enhance human capabilities should be inhibited no matter how safe or efficient. Furthermore, multi-target gene-editing can be developed to achieve a radical cure for polygenic diseases.

## Author Contributions

GC and XX designed the study and wrote the manuscript. RZ revised the manuscript and designed the tables. JH revised the manuscript and painted the figures. HD revised the manuscript and supervised the whole study. All authors contributed to the article and approved the submitted version.

## Funding

This study was supported by National Natural Science Foundation of China (Grant No. 81870354) and Scientific Research Project of Shanxi Provincial Health Commission (Grant No. 2021063).

## Conflict of Interest

The authors declare that the research was conducted in the absence of any commercial or financial relationships that could be construed as a potential conflict of interest.

## Publisher's Note

All claims expressed in this article are solely those of the authors and do not necessarily represent those of their affiliated organizations, or those of the publisher, the editors and the reviewers. Any product that may be evaluated in this article, or claim that may be made by its manufacturer, is not guaranteed or endorsed by the publisher.
